# Advances in the diagnosis and treatment of acute acquired comitant esotropia

**DOI:** 10.1007/s10792-024-03231-5

**Published:** 2024-07-05

**Authors:** Shuyang Guo, Yulian Zhou, Sida Xi, Chen Zhao, Wen Wen

**Affiliations:** 1 Department of Ophthalmology and Visual Science, Eye and ENT Hospital, Shanghai Medical College, Fudan University, Shanghai, 200031 China; 2 NHC Key Laboratory of Myopia, Fudan University, Shanghai, China; 3 Shanghai Key Laboratory of Visual Impairment and Restoration, Fudan University, Shanghai, China

**Keywords:** Acute acquired comitant esotropia, Diplopia, Treatment, Etiology

## Abstract

Acute acquired comitant esotropia (AACE) is mainly characterized by sudden onset, accompanied by diplopia, without extraocular muscles paralysis or ocular motility disorders. In recent years, the incidence of AACE has been increasing, researchers have found that this phenomenon may be related to the widespread use of electronic devices and the increase in the number of people working from home during the COVID-19 pandemic. However, its neural mechanisms have not been fully elucidated. This article primarily reviews the latest developments in the diagnosis and treatment of AACE from the perspectives of etiology and treatment methods, aiming to provide direction for future in-depth exploration of the pathogenesis and treatment approaches of this disease.

## Introduction

Acute acquired comitant esotropia (AACE) is characterized by sudden onset of strabismus, accompanied by diplopia, with no significant changes in the deviation angle in different gaze positions. AACE accounting for 0.3% of strabismus cases in children [1] and it has been reported that older children and adults have been more susceptible to AACE. The incidence of AACE has risen recently, likely due to the increased use of electronic devices and the growth in home-based work prompted by the COVID-19 pandemic [2–4]. Among cases involving refractive errors, myopia is the most common, with 90% of adult AACE patients experiencing myopia [5]. Hyperopia is more common in pediatric AACE patients, which may be related to physiological hyperopia in children [6]. The study by Sturm et al. [7] investigated the clinical characteristics of 24 children with AACE, all of whom were aged three years or younger, with all 24 patients were classified as the Franceschetti type (mentioned in the next paragraph).

## Methods

**Fig. 1:**
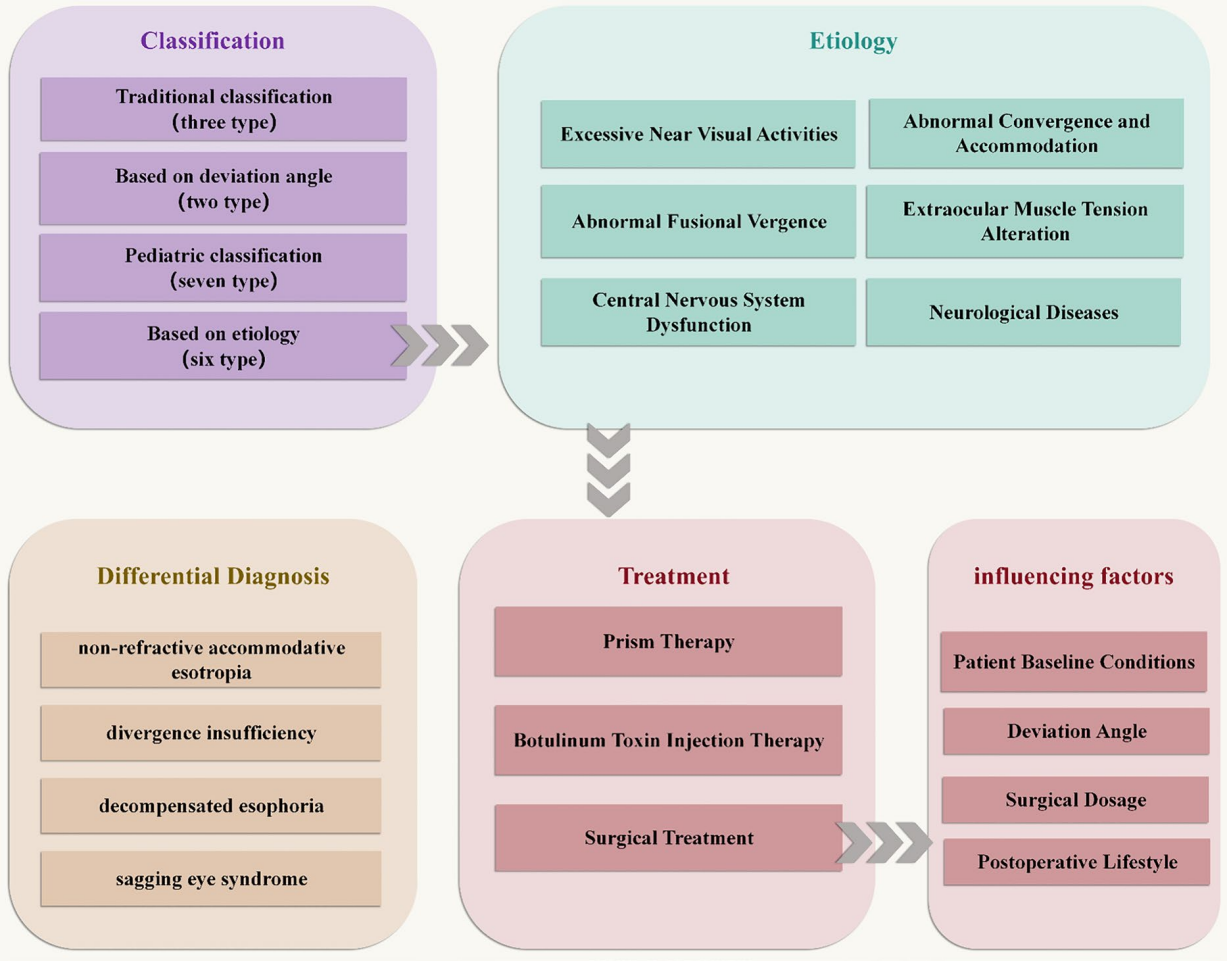
Flowchart about the key concepts and topics in this review

Literature was collected from Web of Science, Medline and Pubmed using the terms: ‘‘acute acquired comitant esotropia’’, “acute onset esotropia”, ‘‘diagnosis’’ and ‘‘treatment’’. By reviewing abstracts, we summarized the classification, etiology, diagnosis, and treatment of AACE, aiming to identify gaps in the field and provide insights for future research and clinical practice (Flowchart see Fig. 1).

### Clinical classification and characteristics

In 1958, Burian et al. first divided AACE into three types [8]. This classification method is still widely used today and is the most wide-used classification in clinics. Type I (Swan type), firstly proposed by Swan in 1947 [9], often develops following monocular occlusion or a decline in monocular vision due to illness, resulting from the disruption of binocular fusion function. Type II (Burian-Franceschetti type) is common in patients with mild hyperopia [7], characterized by a larger degree of strabismus [7] and a smaller accommodative factor [7], this type often occur after severe illness or psychosomatic trauma. Type III (Bielschowsky type) involves patients with myopia (up to 5.00 D), where diplopia occurs at distance but disappears at near. Some scholars believed that there had been no significant distinction between Type II and Type III, with refractive errors primarily associated with patient age, and they propose a classification based on the degree of strabismus which appears to be more effective for clinical diagnosis and treatment [10]. Buch et al. [6], in their research on 48 children with AACE, classified pediatric AACE into seven types, including Occlusion-related AACE; Idiopathic AACE; Acute accommodative AACE; Decompensated AACE; Neurological AACE; Cyclic AACE; Secondary AACE. Notably, the traditional Bielschowsky type was not observed in this study. Additionally, owing to children's physiological hyperopia, hyperopia is comparatively frequent in pediatric AACE patients. However, the controversy of classification of AACE was attribute to unknown etiology.

### Etiology

The specific pathogenesis of AACE remains unclear to this day. Based on a comprehensive analysis of existing reports, the causes of AACE can be categorized into the following types (see Table 1)**.**

**Table 1:** Etiologies of AACE

Etiologies of AACE		Author, year
Excessive near visual activities		Zhu et al. [2], Burian et al. [8], Yagasaki et al. [11], Roda et al. [12], Nouraeinejad [13], Lekskul et al. [14], Ruatta et al. [15]
Abnormal convergence and accommodation	Convergence spasm	Yagasaki et al. [11], Nouraeinejad [13], Campos [18], Kemmanu [19]
	Excessive accommodation	Lee et al. [20]
	Excessive convergence	Tong et al. [21]
Abnormal fusional vergence	Reduced fusional divergence amplitude	Roda et al. [12], Campos [18]
	Inadequate compensatory enhancement of fusional divergence amplitude	Ali et al. [23], Zhao et al. [24]
Extraocular muscle tension alteration	Changes in tension	Burian et al. [8], Erkan et al. [25], Webb et al. [26]
	Changes in position	Cai et al. [31], Hayashi et al. [32]
Central nervous system dysfunction		Hu et al. [37]
Neurological Diseases	Chiari I malformation	Biousse et al. [38], Hentschel et al. [39], Weeks et al. [40]
	Cerebellar astrocytoma	Lee et al. [41], Dikici et al. [42], Musazadeh et al. [43]
	Cerebellar medulloblastoma	Zweifach et al. [44]
	Diffuse intrinsic pontine glioma	Osborne et al. [45]
	Secondary abducens nerve injury	Yatziv et al. [50]

### Excessive near visual activities

Prolonged engagement in close visual activities, such as using electronic devices, studying, assembling or repairing precision equipment, may be a significant risk factor for AACE [2, 8, 11–15]. Excessive near-work activities may lead to increased tension in the medial rectus muscle, thereby disrupting motor fusion; overuse of digital devices at night significantly affects visual motion processing and ocular adjustment, resulting in the breakdown of motor and sensory fusion, thereby triggering the disease [2]. Roda et al., in their study of 117 AACE patients, hypothesized that long-term near-work activities may weaken the ability to relax fusion from near to distance vision, explaining why 94% of the patients experience diplopia at distant viewing points [12]. Studies have found that reducing near-work time helps decrease the degree of strabismus in AACE patients [13, 16, 17]. For patients excessively engaged in near visual activities, methods such as correcting myopia and reducing the use of electronic devices can effectively decrease their daily time spent on near visual activities, thereby reducing the impact of this factor.

### Abnormal convergence and accommodation

Convergence spasm may be a cause of Bielschowsky type and AACE associated with prolonged close-work [11, 13, 18, 19]. In over-corrected myopic patients, convergence spasm may arise from excessive accommodation and convergence while viewing close objects, and spasm do not relax during distant viewing, thus leading to diplopia. Research by Lee et al. indicates that excessive use of electronic devices at close range could result in increased visual accommodation requirements, thereby provoking excessive accommodation and triggering the disease [20]. A study involving 22 AACE patients revealed that their average accommodation convergence/accommodation (AC/A) ratio exceeded normal range, implying that excessive convergence might be a trait of AACE. The study further noted that the accommodation capacity of these patients is below that of age-matched healthy individuals. The deficiency in accommodation leads to increased convergence during close-work activities, resulting in chronic overuse of the medial rectus muscles, upsetting the balance with the lateral rectus muscles, and thus leading to AACE [21].

### Abnormal fusional vergence

Patients with AACE exhibit abnormalities in fusional vergence movements. Swan type and AACE associated long-term occlusion therapy is associated with abnormalities in fusion function [8, 22]. Research by Roda et al. discovered a negative correlation between the deviation angle during diplopia episodes and age, hypothesizing a reduction in fusional reserves with aging. This could account for the occurrence of diplopia in the elderly with smaller esotropia angles [12]. A reduced fusional divergence amplitude may be one of the causes of AACE onset [18].The development of Type II AACE could be associated with a diminished fusional divergence amplitude [18]. In a study involving 8 AACE patients, Ali et al. discovered that their patients did not fit into any type of the traditional classification, indicating that their conditions resulted from decompensation of esophoria due to inadequate compensatory enhancement of fusional divergence amplitude [23]. A prospective study also found fusional vergence dysfunction in adult AACE, with reduced fusional convergence amplitude and increased fusional divergence amplitude, which may be an adaptive phenomenon for esotropia to maintain fusion in AACE patients [24]. Both reduced and increased fusional divergence amplitudes have been found in AACE, because their etiology is different. The increased fusional divergence amplitudes acts as a compensatory factor. The decreased fusional divergence amplitudes is due to excessive near vision activities or reduced fusional reserves. In AACE, fusional convergence is related to the degree of myopia, the more severe the myopia, the greater the fusional convergence in AACE.

### Extraocular muscle tension alteration

Changes in the tension and position of the medial rectus muscle may be a factor leading to AACE. Researchers have proposed that excessive close-range visual activities might heighten the muscular tension in the medial rectus muscles, simultaneously reduce the tension in the lateral rectus muscles [8, 25, 26] and cause lateral rectus atrophy [27]. Magnetic resonance imaging(MRI) can be used to observe the morphological and positional changes of the extraocular muscles in strabismus patients, providing a reference for subsequent MRI studies on the changes in the extraocular muscles in AACE [28]. Research has discovered that in patients with AACE, there are significant changes in the muscle size and volume in the dominant eye, which are adaptations to their visual problems and solutions to the issue of double vision [29]. A Study indicates that the insertion site of the medial rectus muscle remains the most consistent across all extraocular muscles, irrespective of the patient's ethnic background [30]. Some research has found that in AACE patients, the distance from the conjunctival insertion point of the medial rectus muscle to the limbus is relatively short, which may enhance convergence ability, disrupt the balance between convergence and divergence, and subsequently lead to AACE [31, 32].

### Central nervous system dysfunction

Previous studies have found abnormalities in brain functional areas in patients with non-AACE strabismus. Studies on animal models of strabismus have shown a decrease in the number of binocular neurons in the V1 and V2 areas of the visual cortex, and these neurons can’t regenerate once lost, even if binocular vision returns [33]. Binocular neurons are fundamental to the development of stereopsis [34], which helps explain why strabismus patients often exhibit binocular visual impairments. Research on strabismic cat models found that strabismus affects the internal and inter-regional neural connections within the cat's visual cortex. Strabismus leads to the segregation of ocular dominance domain in the primary visual cortex (area 17) and alters the connections within area 18 and between areas 17 and 18 [35]. Studies on strabismic monkey models have found that the disruption of binocular fusion may lead to connection defects in the V1 area and potentially induce esotropia [36]. Scholars have used resting-state functional magnetic resonance imaging (rs-fMRI) to investigate cortical abnormalities in AACE patients [37]. This study collected and analyzed rs-fMRI signal data from 25 AACE patients and a matched control group of 25. Compared to the control group, the primary visual cortex and dorsal pathway in AACE patients exhibited significant changes in the amplitude of low-frequency fluctuations (ALFF), and these changes were correlated with clinical characteristics such as age of onset, time spent on near work activities, and deviation angle of strabismus. These findings not only reveal the connection between AACE and the visual center but also provide new clues for understanding the pathogenesis and therapeutic approaches of AACE.

### Neurological diseases

The appearance of AACE may associate with neurological disease, include Chiari I malformation [38–40], cerebellar astrocytoma [41–43], cerebellar medulloblastoma [44], diffuse intrinsic pontine glioma [45]. Some scholars believe that intracranial diseases causing AACE are due to lesions affecting the vergence mechanism of midbrain [46, 47]. Others suggest that combined damage to the thalamus and midbrain can affect convergence function, leading to the occurrence of AACE [48]. One study found that patients developed AACE after receiving high doses of carbamazepine, which resolved after dose reduction. However, this is the only resarch suggesting a connection between AACE and medication, indicating a need for further research to explore this association [49]. A study by Yatziv et al. found that epidural puncture decreases intracranial pressure, causing stretching injury to the abducens nerve, leading to comitant esotropia [50].

There is still controversy regarding whether AACE patients require neurological examinations. Some scholars suggest a comprehensive neurological assessment for AACE patients [19], especially in cases with nystagmus or other abnormal ocular and neurological signs (such as cerebellar ataxia, papilledema and so on) [10, 51–54]. Some scholars believe that the possibility of intracranial disease should still be considered even if the patient does not exhibit other neurological and ocular symptoms [55]. Buch et al. [6], based on their study of 48 children, found that 6% cases involved intracranial diseases. They proposed four risk factors for pediatric AACE: neurological symptoms, large deviation angle at distance, recurrent AACE, and older age at onset. These risk factors can guide clinicians in deciding when to perform brain imaging. Patients without neurological and ocular abnormal manifestations have a low likelihood of underlying intracranial disease [56, 57]. When treating AACE, the most important consideration is that AACE may be the initial symptom of a neurological disorder. Since most of the neurological conditions are serious, patient must undergo immediate imaing, followed by close monitoring and assessment for signs of intracranial disease [58–60].

The angle of deviation at near and distance fixation also varies among AACE patients. For most patients, the angle of deviation is greater at distance than at near, which may be related to divergence insufficiency [61], excessive near vision activity [62], higher divergent fusional amplitudes at near [15], and intracranial pathological causes. As the disease progresses, the angles of deviation at near and distance tend to become similar. There are also patients whose angle of deviation at near is greater than at distance, which may be associated with convergence excess-induced AACE.

### Treatment and prognosis

There are currently no explicit guidelines for the clinical treatment of AACE, and its treatment methods are still a subject of debate. AACE does not resolve spontaneously, and the vast majority of patients require treatment. Treatment methods for AACE include refractive errors correction, medication [63, 64], occlusion therapy, pharmacological therapy [65], prism therapy, botulinum toxin injections, and surgery. It has been reported that eliminating environmental factors can lessen the severity of esotropia [13, 16, 17]. Environmental factors, like the excessive use of mobile phones and digital screens at close range, must be considered before formulating a treatment strategy. Vision correction also need to take into consider, it is a prerequisite before implementing other treatments [54].

#### Differential diagnosis

AACE should be separate from the non-refractive accommodative esotropia(also named convergence excess esotropia) [66–68], divergence insufficiency [69–73] and decompensated esophoria [74, 75].

We summarize the characteristics (such as deviation angle, diplopia, prism adaptation test, AC/A ratio, fusional amplitudes) of AACE [15, 21, 24, 76] and the terms mentioned above (see Table 2).

**Table 2:** Characteristics about divergence insufficiency, convergence-excess esotropia, decompensated esophoria and AACE

	APCT	Diplopia	Prism adaptation test	AC/A ratio	Fusional amplitudes	NPC	NPA	comitance versus incomitance
Divergence insufficiency	Distance fixation: esotropia; near fixation: orthophoria; distance > near	Distance > near	NA	Low or normal	Fusional divergence: Low	NA	NA	Comitance
Convergence-excess esotropia	Near fixation: > 10PD; distance fixation: orthophoria; distance < near	Distance < near	Reveal a large deviation angle at distance (> 10-20PD)	High	NA	NA	Low or normal	Comitance
Decompensated esophoria	Near fixation: esotropia; distance fixation: esotropia; similar	NA	Increased significantly	NA	NA	NA	NA	Comitance
AACE	Distance fixation: esotropia; near fixation: orthophoria; distance > near	Distance > near	Increased significantly	High	Fusional convergence: Low fusional divergence: High	NA	NA	Comitance

*NA* not available, *APCT* alternate prism cover test, *PD* prism diopters, *NPC* near point convergence, *NPA* near point accommodation

It is worth noting that there is an overlap between AACE and convergence excess esotropia, divergence insufficiency, and decompensated esotropia. Based on the above analysis of etiology, it can be observed that some AACE patients may exhibit characteristics such as excessive convergence, reduction in fusional reserves, and reduced fusional divergence amplitude.

AACE also need to seperate from sagging eye syndrome, wich can also present with distance esotropia. We can differentiate based on characteristics such as cyclovertical strabismus, limited supraduction, and blepharoptosis [77].

#### Prism therapy

For patients with esotropia less than 15PD, prism therapy can be considered [54]. For strabismus cases not exceeding 25 PD, progressive prism therapy has demonstrated positive results in accommodative movement and binocular vision [78].

#### Botulinum toxin injection therapy

Botulinum toxin therapy involves injecting botulinum toxin into the medial rectus muscle to weaken its strength and thus improve the condition of strabismus. There are several methods for injecting BTXA, including microinjection through a small conjunctival incision [79, 80], electromyography(EMG) guided injection without a conjunctival incision [81], and direct conjunctival injection without an incision or EMG [79, 82–87]. With advantages like minimal invasiveness, simplicity, low cost, and rapid recovery, BTXA treatment is still a feasible choice for patients with minor strabismus angles and those who urgently require treatment with an unstable strabismus degree for less than six months.

The metabolic characteristics of botulinum toxin indicate that the efficacy of the treatment can be ascertained only after several months. Studies show that the residual angle of deviation and success rate six months after botulinum toxin treatment are related to the angle of deviation two weeks into the treatment. Thus, the deviation angle at two weeks can serve as an indicator of early treatment effectiveness, predicting long-term efficacy [82]. Botulinum toxin treatment is dose-dependent, so selecting the appropriate injection dose based on the angle of strabismus can enhance the success rate of the treatment. Xu et al. [33] selected injection doses according to the degree of strabismus, achieving a six-month follow-up success rate of 86.2% and a final follow-up success rate of 82.2% [79].

We have compiled a table summarizing research on the use of botulinum toxin in AACE, with studies meeting the following criteria: published within the last decade, at least six months follow-up, and a single botulinum toxin injection. The aim is to investigate the effectiveness and feasibility of botulinum toxin in the management of AACE from a quantitative perspective (see Table 3) [79, 80, 82–87].

**Table 3:** Clinical trials investing the efficacy of botulinum toxin in AACE

Study	No. of pts	Age(years)	Type. BX	Dose(IU)	Methods	Follow-up(mos)	Pre-injection angle(PD)	Post-injection angle(PD)	Definition of success	Success rates	Complications
Xu et al. [79]	29	14.2 ± 7.4[4–34]	BTXA (Hengli, China)	15-20PD 2.5U(Uni) 20-40PD 5.0U(Bi) 40-60PD 6.0U(Bi)	S2	6	Distance 40.2 ± 17.7 near 38.4 ± 18.9	Distance 3.0 ± 5.9 near 0.6 ± 4.1	Deviation ≤ 10PD evidence of binocular single vision	86.2%(25/29)	Transient exotropia 24%(7/29) transient hypertropia 14%(4/29) transient ptosis 10%(3/29)
Li et al. [82]	51	23.9[6–50]	BTXA (Hengli, China)	10-25P 3.0U 26-35P 3.5U > 35PD 4.0U	S1	6	Distance 20[15–30] near 18[12.5–25]	Distance 6[4–14] near 4 [2–8]	Deviation ≤ 8PD diplopia disappeared	58.8% (30/51)	Transient ptosis transient exotropia
Wan et al. [83]	16	3.1[2.2–9.3]	BOTOX (Allergan, US)	5.0U(Bi) 1 patients 45PD 3.5U(Bi) 1 patients 40PD 4.0U(Bi) (1U/0.01ml)	S1	6	35[10–55]	0 [0–18]	Deviation ≤ 10PD evidence of binocular single vision no need for retreatment	81%(13/16)	Transient ptosis 50%(8/16) transient exotropia 56%(9/16)
Shi et al. [84]	40	3.1[2.2–9.3]	NA	< 30PD 4U(Bi) 30-40PD 5U(Bi) 40-50PD 6U(Bi) > 60PD 7U(Bi)	S1	6	Distance 32.3 ± 15.4 near 27.1 ± 13.8	5.53 ± 2.52	Deviation ≤ 10PD diplopia disappeared	72.5%(29/40)	Transient ptosis 17.5%(7/40)
Lang et al. [85]	13	12.61 ± 6.74[3–24]	BTXA (Hengli, China)	2.5U(Uni) (1U/0.04ml)	S1	6	Distance 47.30 ± 22.69 near 41.92 ± 27.65	Distance 6.84 ± 6.28 near 5.92 ± 7.72	Deviation ≤ 10PD	84.6%(11/13)	Transient ptosis 15.4%(2/13)
Yu et al. [87]	73	29.88 ± 11.18	NA	10-15PD 3.0U 16-20PD 3.5U 21-40PD 4.0U 40-50PD 4.5U	S1	6 23.27 ± 1.17	Distance 25.86 ± 13.38 near 24.51 ± 13.31	NA	Deviation ≤ 5PD diplopia disappeared	NA cumulative non-recurrence rate: 6mos 90.41% 1yr 82.19% 2yrs 68.68%	Transient exotropia 2.74%(2/73) transient ptosis 6.85%(5/73)
Suwannaraj et al. [86]	34	13.5 ± 10.5	BOTOX (Allergan, US)	5U(Uni) (1U/0.02ml)	S1	6	51.2 ± 14.3	31.3 ± 23.8	Deviation < 10PD	26.5%(9/34)	Transient ptosis 32.4%(11/34) transient exotropia 5.9%(2/34)
Huang et al. [80]	33	30.09 ± 10.94[16–55]	BOTOX (Allergan, US)	< 20PD 1.0–2.5U 20-40PD 2.0–4.0U > 40PD 4.0–6.0U	S2	6	Distance 24.76 ± 6.43 near 20.24 ± 6.80	Distance 7.30 ± 6.17 near 5.15 ± 5.85	Deviation ≤ 10PD diplopia disappeared	87.9%(29/33)	Subconjunctival hemorrhage 6.07%(2/33) limited eye movement 9.09%(3/33) mild vertical strabismus 3.03%(1/33)

*NA* not available, *Uni* unilateral, *Bi* bilateral, *APCT* alternate prism cover test, *PD* prism diopters, *mos* months, *yr* year, *S1* injection without conjunctival incision and EMG guidance, *S2* injection with conjunctival incision and without EMG guidance

There is still no definitive conclusion regarding the comparative effectiveness of botulinum toxin treatment versus surgery, with some studies indicating similar therapeutic outcomes for both approaches [83–85, 88, 89]. Yu et al. compared the effects of BTXA injections with high-dose surgical treatment, and their two-year follow-up found that the recurrence rate in the BTXA group was significantly higher than that in the surgical group [87]. Suwannaraj et al. [86] also concluded that surgical treatment is more effective than botulinum toxin injections, but this conclusion is limited as all patients received the same dose of BTXA, without considering the dose-dependent effects of botulinum toxin, thus limiting the reference value of their conclusion.

#### Surgical treatment

Surgical therapy primarily aims to correct strabismus and eradicate diplopia. Surgical intervention is essential for patients with significant strabismus. Common surgical procedures include bilateral/unilateral medial rectus recession (MRrec) [11] and unilateral MRrec + unilateral rectus resection (LRres) [5, 90, 91]. Currently, there is no clear evidence indicating the difference in efficacy between these two surgical methods, so surgeons usually select the surgical approach based on their clinical experience. Some scholars believe that for patients with a large angle of strabismus, unilateral MRrec + unilateral LRres is more effective than bilateral/unilateral MRrec, and shortening the lateral rectus muscle can effectively reduce the risk of postoperative recurrence and leave a fresh eye, which may be beneficial for possible future surgical needs [92]. Methods to evaluate surgical outcomes include assessing residual strabismus and its severity, recurrence of diplopia [12], restoration of stereopsis [93], and the balance in visual dominance [94]. The effectiveness of surgical treatment is influenced by various factors, which will be explained in the following sections.

Patient baseline conditions: Better vision acuity can help improve postoperative sensory fusion; The success rate of surgery in elderly patients may be reduced due to factors such as degeneration of periocular tissues [12].

Angle of deviation: Surgical is most appropriate when the deviation angle stabilizes following at least six months conservative treatment [54].

Selection of Surgical Dosage: In AACE surgical treatment, choosing the appropriate surgical dosage is a key focus, but there are currently no definitive guidelines for selecting surgical dosages in AACE. Many scholars suggest increasing the dosage of bilateral medial rectus recession (BMR) surgeries [12, 23, 95, 96]. In eight cases of BMR surgery performed by Ali et al. based on Park's surgical table, five cases (71%) had residual strabismus of 2PD-14PD postoperatively [23]. Lee et al. [96] performed augmented-dosage BMR surgeries on 16 patients, achieving a postoperative success rate of 75%. Regarding unilateral MRrec + unilateral LRres(RR) surgery, Zhou et al. [92] proposed that the RR surgical dosage for AACE patients should be greater than that for typical esotropia patients, and they were the first to propose the dose–response reference of RR in AACE. For patients with a deviation of less than 30 diopters, the dose–response ratios for MRrec and LRres are 5.11 PD/mm and 2.51 PD/mm, respectively. In cases where the deviation is greater than or equal to 30 diopters, an additional LRres has a dose–response ratio of 5.48 PD/mm to address the excess deviation over 30 diopters. Further research has introduced the dose–response reference for MRrec surgery, like Zhou's approach, using 30PD as the cut-off point [87]. The prism adaptation test(PAT) can reveal the potential angle of strabismus, and using this angle as the surgical target can help improve binocular function and reduce the recurrence rate of AACE [15, 54, 97]. However, PAT requires patient highly compliance, limiting its clinical application value. A study used the preoperative Base-out (BO) recovery point as the target surgical amount, and the results showed that 86.4% patients maintained normal eye position during an average follow-up period of 15.8 months [98]. Compared to PAT, this measurement method is simpler and more convenient, demonstrating higher clinical application value. Research also indicates that using the maximum angle of strabismus measured after one hour of occlusion as the surgical target (with 14 patients showing an increase in esodeviation of more than 5 prism diopters after occlusion), combined with the non-adjustable suture technique, resulted in favorable outcomes [99]. Another study [100] compared the treatment effects using Maddox rod prism degrees and prism and alternate cover test(PACT) degrees as target amounts for AACE surgery. Data analysis revealed that, The Maddox rod prism method is capable of detecting a higher degree of strabismus in patients with AACE. Utilizing this measurement as a target metric significantly reduces residual strabismus and increases the success rate of surgical interventions. Consequently, it is advisable to incorporate the Maddox rod prism as a standard preoperative assessment technique for patients with AACE.

Postoperative Lifestyle: The use of electronic devices should be appropriately limited after surgery. This is particularly important for younger patients who are unable to manage their time spent on electronic devices responsibly, leading to a higher probability of recurrence and double vision post-surgery compared to adults. For these patients, strict management of electronic device usage time is necessary [101].

Patients with AACE have the potential to recover normal stereopsis [10, 93]. The time from the onset of the condition to the start of treatment does not seem to be a critical factor in the recovery of normal stereopsis [61, 93, 102]. Another study has discovered that improvements in the angles of esotropia are negatively correlated with the length of untreated time, highlighting the significance of early intervention to avert further deterioration of esotropia [65]. Sturm et al., based on a study of 22 Type II AACE patients, found that the interval between the onset of esotropia and surgery was similar among patients with normal stereopsis and those with absent or lower levels of stereopsis (23 months compared to 24 months), the study also found that the recovery interval for stereopsis was surprisingly long, with the authors suggesting a correlation with patients who had undiagnosed monocular fixation syndrome [93]. Subsequent research revealed that Bagolini striated lenses and the Worth four-dot test could differentiate between AACE and monocular fixation syndrome [103]. In observing the postoperative outcomes of AACE, Kim et al. [90] considered patients with unrecovered stereopsis to have uncompensated monocular fixation syndrome.

## Conclusions and future perspectives

AACE has seen an increase in research in recent years due to the rising incidence of this condition. Currently, research on AACE still faces several knowledge gaps: the classification, causes of the disease, choices of treatment methods, and factors affecting treatment efficacy. People with AACE have many problems such as convergence, accommodation, and fusional amplitudes. These patients should receive a comprehensive examination including measurements of deviation angles at near and distance fixation, fusional amplitudes, AC/A ratio, near point accommodation (NPA), near point convergence (NPC), and accommodative facility.

Future research needs to delve deeper into the etiology of AACE, especialy with advanced neuroimaging techniques such as Electroencephalograph (EEG), fMRI. More RCT studies need to perform in preferred treatment in small-angle AACE to form guideline of AACE treatment.

## Abbreviations

AACE: Acute acquired comitant esotropia
AC/A: Accommodation convergence/accommodation
MRI: Magnetic resonance imaging
rs-fMRI: Resting-state functional magnetic resonance imaging
ALFF: Amplitude of low-frequency fluctuations
EMG: Electromyography
MRrec: Medial rectus recession
LRres: Lateral rectus resection
BMR: Bilateral medial rectus recession
RR: Unilateral medial rectus recession + unilateral Lateral rectus resection
PAT: Prism adaptation test
BO: Base-out
PACT: Prism and alternate cover test
EEG: Electroencephalograph
NPA: Near point accommodation
NPC: Near point convergence
